# Analysis of the Content of Cadmium and Zinc in Parts of the Human Hip Joint

**DOI:** 10.1007/s12011-014-0168-4

**Published:** 2014-11-15

**Authors:** Barbara Brodziak-Dopierała, Jerzy Kwapuliński, Krzysztof Sobczyk, Danuta Wiechuła

**Affiliations:** 1 School of Pharmacy, Division of Laboratory Medicine, Department of Toxicology, Medical University of Silesia, 4 Jagiellonska, Str, 41-200 Sosnowiec, Poland; 2 Institute of Occupational Medicine and Environmental Health, 13 Kościelna, Str, 41-200 Sosnowiec, Poland; 3 Department of Traumatic Surgery, Municipal Hospital, May-1 Str, 41-100 Siemianowice Slaskie, Poland

**Keywords:** Cadmium, Zinc, Hip joint, Bone, AAS

## Abstract

Cadmium is an element with proven direct and indirect toxic effects on bones. Zinc affects the content of cadmium in the human body. These elements show antagonistic interactions. The aim of the research was to determine the levels of cadmium and zinc in the hip joint tissues and interactions between these elements. The study group consisted of 91 subjects, 66 women and 25 men. The tissues were obtained intraoperatively during hip endoprosthetic surgery. The levels of cadmium and zinc were assayed by the atomic absorption spectrophotometry (AAS) method. The analysis of the content of cadmium and zinc in different parts of the hip joint, i.e., articular cartilage, cortical bone, and cancellous bone of the femoral head as well as the articular capsule and a fragment of the cancellous bone taken from the intertrochanteric region of the femoral bone showed significant differences. The cancellous bone was found to have the highest potential to accumulate the elements studied, whereas part of the articular capsule the lowest. Higher levels of cadmium and zinc were observed in samples obtained from men. Patients with bone fractures had higher cadmium content than those with osteoarthritis. The study on the content of cadmium and zinc in the tissues of the hip joint is one of the primary research biomonitoring.

## Introduction

Reconstruction processes that occur in bone tissues allow its proper functioning, cause inclusion of additional elements, also toxic, in the reconstructed bone, and affect metabolic processes in the bone, which may lead to disorders in the osteoarticular system manifested as changes within bone tissues and other organs.

Cadmium shows a negative effect on bone tissues. However, its mechanism is not fully understood. One of the possible actions involves interaction between cadmium and calcium, which results in calciuria and leads to a reduction in calcium absorption from intestines. Moreover, a relationship has been shown between the increased content of cadmium in the body and a decreased level of mineral density of the bones, increased bone resorption and decreased levels of the parathyroid hormone. This metal influences trace elements that affect bone tissue, mainly due to the interaction among zinc, iron, and copper [1].

The level of cadmium accumulated in bones can affect the activity of bone cells and directly increase the loss of mineral elements in the bones. Furthermore, the results indicate that this metal activates osteoclasts and inhibits osteoblasts [2].

Long-term exposure to cadmium can result in skeletal disorders, i.e., osteoporosis or osteomalacia with an increased number of bone fractures. It has been observed that exposure to cadmium affects bone metabolism directly and indirectly, reducing the content of minerals and bone matrix [3].

Chronic cadmium intoxication in animals reduces bone mineral density (BMD) and mechanical endurance, decreases the content of inorganic elements that are largely responsible for the trabecular bone structure, and decreases the activity of alkaline phosphatase [4]. The toxic activity of cadmium is not only limited to bone tissue quantity reduction but also to its quality deterioration. Cadmium intoxication results in a decreased level of types I and V collagen in the bones and leads to their increased solubility by damaging the intermolecular cross-links [5]. A change in the collagen structure decreases its endurance, thus increasing susceptibility to deformation and fractures. Chronic exposure to cadmium compounds causes histopathological changes in the bone structure manifested by thinning of the metaphyseal cortical bone and expanding Haversian canals which are filled with the mineralized bone matrix. Osteocytes become rounded and spaces between them widened. Also, bone marrow hyperplasia occurs [6].

The toxic effect of cadmium compounds on bone tissue is associated with kidney damage, secondary metabolic vitamin D disorders, and an increase in renal excretion of calcium, a decrease in intestinal absorption of calcium, and calciotropic hormone disorders. The accumulation of cadmium in proximal tubular cells reduces the conversion of 25 (OH) D into 1,25 (OH_2_) D_3_. Reduction in the level of active 1,25 (OH_2_) D_3_ decreases the absorption of calcium in the small intestine and the bone tissue mineralization process, contributing to osteomalacia [7, 8]. Another mechanism of the toxic activity of cadmium involves blocking the insulin-like growth factor (IGF) production stimulated by zinc and, consequently, reducing the production of bone matrix proteins by osteoblasts, mainly in older cell populations. This may lead to a toxic effect of cadmium on bone tissue in later years of life [9].

The content of cadmium in the body is usually higher in women than in men due to increased gastrointestinal absorption at lower iron concentrations [9].

Cadmium and zinc show antagonistic interactions. Cadmium ions inhibit the intestinal absorption of zinc. It has been concluded that the simultaneous administration of cadmium and zinc to experimental animals reduces cadmium intoxication symptoms as compared with cadmium given alone [10].

Zinc is an essential element for proper functioning of the human body, with many important metabolic functions. For example, zinc protects the body against free radicals, stimulates metallothionein synthesis, stimulates proliferation and differentiation of osteoblasts, and regulates the activity of vitamin D. Moreover, it prevents bone resorption that is stimulated by the parathyroid hormone. Zinc deficiency or its excessive loss by kidneys can lead to osteoporosis. Research studies on experimental animals kept on a low-zinc diet have shown that there is a reduction in the size and length of bones, they become more prone to fracture, and the quantity of trabecular bones decreases [10, 11].

Furthermore, zinc plays an important role in the process of bone mineralization. It stimulates synthesis and enhances the effect of many growth factors, such as IGF-1, on hard tissue. Bone tissue growth is stopped by zinc deficiency. Bones become thin and brittle, and severe resorption occurs [12].

The heads of the femur and the surrounding tissues can be obtained for research purposes due to the ever-increasing number of hip endoprosthetic procedures. This allows the analysis of the content of selected metals and assessment of the influence of their accumulation on degenerative processes and bone fracture. The aim of the current study was to determine the levels of cadmium and zinc in different parts of the hip joint in men and women. Moreover, the study investigated the coexistence of Cd and Zn in different parts of the hip joint with respect to patients’ gender and cigarette smoking. The levels of Cd and Zn in the tissues studied were compared depending on the indications for prosthetic surgery: degenerative changes or fractures of the femoral neck.

## Materials and Methods

The study material included parts of the hip joint obtained from patients inhabiting urban areas of the Upper Silesian Industrial District. The tissue material was collected in Town Hospital in Siemianowice Slaskie, intraoperatively, during hip replacement procedures, according to the consent of the Bioethics Committee. The study was performed from 2008 to 2012. Recommendations for this type of surgery were, in most cases, degenerative changes of the hip.

The analysis involved:

1. Femoral head excised in situ.
2. Anterolateral aspect of the joint capsule, which was routinely excised in order to open the hip joint during surgery.
3. A box-shaped fragment of the cancellous bone from the intertrochanteric area (this fragment was routinely chiseled out from the femoral bone as the initial preparation of the proximal femur before prosthetic stem implantation).

The femoral heads were debrided from residual soft tissues. Fragments of the joint capsule, ligament of the head of the femur, and the femoral neck (especially the medial calcar) were removed with various instruments, such as Liston bone cutting forceps, Luer bone rongeur, and bone curette. In the next stage, bone curette and Luer rongeur were used to remove the articular cartilage and then the subchondral bone until a rounded “core” was obtained made of the cancellous bone only. In some patients, the cartilage was absent or scarce due to progression of osteoarthritis. The subchondral layer of the bone had various widths: from virtually non-existent to a fraction of a millimeter to a few millimeters of the eburniated bone in advanced coxarthrosis. The remaining part of the femoral head consisted mostly of the cancellous bone, sometimes with heterogeneous microarchitecture, with areas of osteosclerosis and geodes filled with fibrous connective tissue. The collected samples were placed in polyethylene bags, labeled, and stored in a freezer at a temperature of −20 ± 1 °C.

In total, 91 hip joint samples were taken, 66 from women and 25 from men. The mean age was 65.7 ± 10.5 years; 67.3 ± 8.6 in women and 61.4 ± 13.6 in men. Two groups were formed in the study population, depending on the indication for endoprosthetics. Group I included patients with fractures of the femoral neck (*n* = 7), and group II with degenerative changes of the hip joint (*n* = 84). The study population consisted of smokers (*n* = 17) and non-smokers (*n* = 74). The non-smokers were mainly women (*n* = 58), with only 16 men. In the group of smokers, there were slightly more men (*n* = 9) than women (*n* = 8).

In order to determine the content of the elements in the femoral bone head using the atomic absorption spectrometry (AAS) method, the samples of known weight were gradually ashed to constant weight in a muffle furnace at a temperature of 100 °C (initially) and 420 °C (later). The analytical sample of ash (approximately 1 g) was digested in 2 cm^3^ of spectrally pure HNO_3_ (V; Supra pure) by Merck (Darmstadt, Germany). The formed solution was transferred into a flask with a volume of 25 cm^3^, and then filled up with distilled water to the scale mark. In the samples prepared in this way, the manganese and iron content was determined using the AAS method and a Pye Unicam SP9 apparatus (Philips ASS, Cambridge, UK). The correctness of the applied methodology was tested using the method of standard addition.

Statistical calculations were made using the Statistica software for Windows 10. The Mann–Whitney *U* test (*p* < 0.01) was used. The differences were considered significant at *p* ≤ 0.50. The Spearman’s correlation was used to determine the relationship between cadmium and zinc (*p* < 0.05).

## Results

The analysis of the content of cadmium and zinc in various parts of the hip joint, i.e., the articular cartilage, the cortical bone, the cancellous bone of the femoral head, the articular capsule, and a fragment of the cancellous bone taken from the intertrochanteric region of the femoral bone showed significant differences. The mean content of cadmium in the respective parts of the hip joint was as follows (μg/g): articular cartilage, 0.81; cortical bone, 0.52; cancellous bone, 0.94; cancellous bone from the intertrochanteric region of the femoral bone, 0.74; and articular capsule, 0.30. The mean content of zinc (μg/g) was: articular cartilage, 50.68; cortical bone, 79.99; cancellous bone, 158.27; cancellous bone from the intertrochanteric region of the femoral bone, 52.60; and articular capsule, 25.47.

**Fig. 1:**
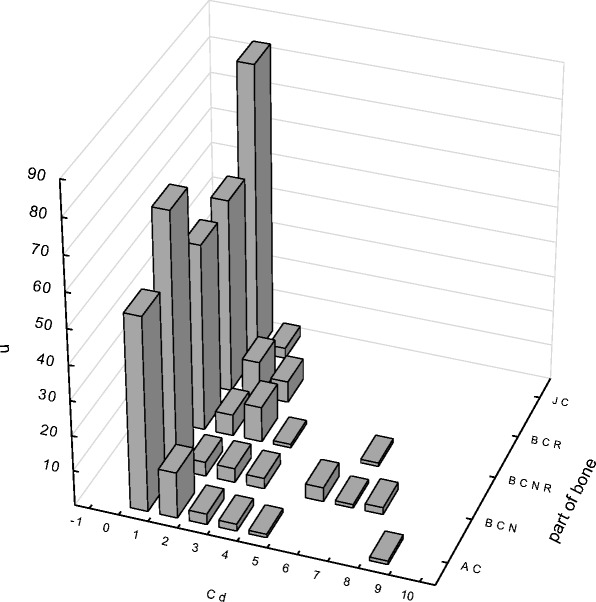
The prevalence content of cadmium in selected parts of the hip joint (μg/g). *AC* articular cartilage, *BCN* cancellous bone, *BCNR* cancellous bone from the intertrochanteric region, *BCR* cortical bone, *JC* joint capsule

**Fig. 2:**
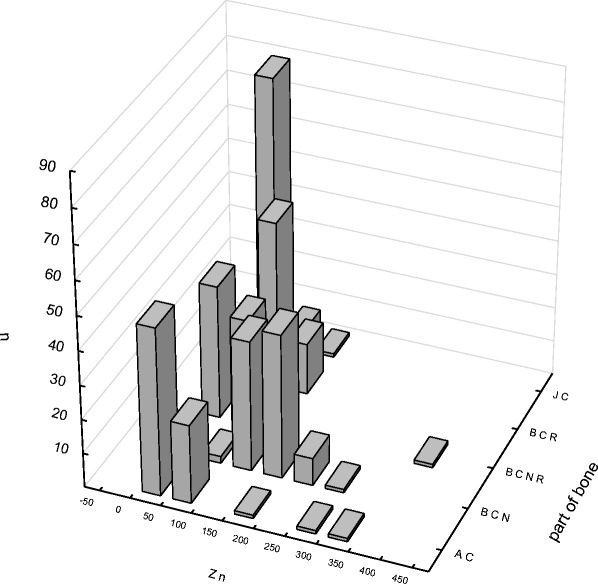
The prevalence content of zinc in selected parts of the hip joint (μg/g). *AC* articular cartilage, *BCN* cancellous bone, *BCNR* cancellous bone from the intertrochanteric region, *BCR* cortical bone, *JC* joint capsule

In various parts of the hip joint, distribution of cadmium and zinc concentrations was similar to normal—righthanded (Figs. 1 and 2).

The highest cadmium content was observed in cancellous tissue, both in women and men (0.85 and 1.17 μg/g), whereas its lowest level was found in a part of the articular capsule in men 0.21 μg/g (Table 1). The content of zinc in the respective parts of the joint was the highest in the cancellous bone and the lowest in the articular capsule. The levels of zinc in the cancellous bone of women and men were, respectively, 155.85 and 165.35 μg/g, and in the articular capsule 26 and 24 μg/g (Table 2). The levels of cadmium and zinc in the respective tissues of the hip joint showed no statistically significant differences within the two genders (Mann–Whitney *U* test, *p* < 0.05).

**Table 1:** Statistical characteristics for concentration of cadmium in parts of the hip joint (μg/g)

	AC		BCR		BCN		BCNR		JC	
	*n* = 51	*n* = 24	*n* = 50	*n* = 21	*n* = 66	*n* = 25	*n* = 49	*n* = 22	*n* = 62	*n* = 24
Women										
AM ± SD	0.78 ± 1.38		0.46 ± 0.73		0.85 ± 1.70		0.62 ± 0.98		0.33 ± 0.32	
Med	0.32		0.1		0.15		0.11		0.21	
Range	0.01–8.21		0.01–2.39		0.01–7.45		0.01–3.73		0.05–1.78	
CV	175		158		200		158		101	
Men										
AM ± SD		0.85 ± 0.78		0.67 ± 0.83		1.17 ± 1.82		1.00 ± 1.57		0.21 ± 0.15
Med		0.62		0.14		0.27		0.13		0.15
Range		0.01–3.25		0.01–2.43		0.01–7.52		0.01–6.66		0.07–0.58
CV		92		125		156		157		70

*AC* articular cartilage, *BCR* cortical bone, *BCN* cancellous bone, *BCNR* cancellous bone from the intertrochanteric region, *JC* joint capsule, *AM* arithmetic mean, *SD* standard deviation, *MED* median, *CV* coefficient of variation

**Table 2:** Statistical characteristics for concentration of zinc in parts of the hip joint (μg/g)

	AC		BCR		BCN		BCNR		JC	
	*n* = 51	*n* = 24	*n* = 50	*n* = 21	*n* = 66	*n* = 25	*n* = 49	*n* = 22	*n* = 62	*n* = 24
Women										
AM ± SD	52.79 ± 55.53		78.98 ± 24.99		155.58 ± 35.47		48.22 ± 17.40		26.15 ± 17.55	
Med	36.32		80.03		157.29		45.94		21.16	
Range	7.95–309.52		14.34–136.38		58.89–292.56		6.28–87.91		6.95–07.82	
CV	105		32		23		36		67	
Men										
AM ± SD		46.21 ± 20.32		82.40 ± 24.86		165.35 ± 38.20		62.33 ± 74.47		23.73 ± 17.62
Med		42.57		78.39		176.17		45.66		20.54
Range		18.27–86.83		49.12–130.92		81.48–222.61		27.48–390.66		5.05–81.85
CV		44		30		23		119		74

*AC* articular cartilage, *BCR* cortical bone, *BCN* cancellous bone, *BCNR* cancellous bone from the intertrochanteric region, *JC* joint capsule, *AM* arithmetic mean, *SD* standard deviation, *MED* median, *CV* coefficient of variation

The comparison of the mean cadmium content in all studied parts of the hip joint revealed its higher level in patients with fractures, i.e., 0.85 μg/g as compared with 0.64 μg/g in patients with degenerative changes. Differences between the levels were statistically significant at *p* ≤ 0.025 (Mann–Whitney *U* test). The highest content of cadmium in both study groups was in the cancellous bone: 1.38 μg/g for fractures and 0.90 μg/g for degenerative changes. The lowest content in both groups was found in the articular capsule: 0.34 μg/g for fractures and 0.30 μg/g for degenerative changes. For the content of zinc, the differences in the group of patients with fractures (67.47 μg/g) and degenerative changes (76.45 μg/g) were not statistically significant (Mann–Whitney *U* test, *p* > 0.10). Like for cadmium, the highest content of zinc was in the cancellous bone and the lowest in the articular capsule.

The content of cadmium in the population of smokers and non-smokers differed significantly (Mann–Whitney *U* test, *p* > 0.01). The level was higher in the group of smokers (0.89 μg/g) than in non-smokers (0.61 μg/g). However, the content of zinc in both populations was almost identical (75.62 and 75.82 μg/g).

**Fig. 3:**
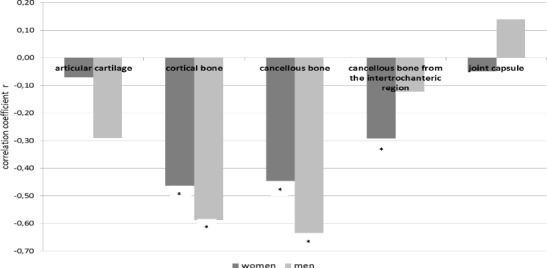
Correlations of cadmium from zinc in selected elements of hip men and women; *p < 0.05, statistically significant

Cadmium and zinc are elements of proven antagonistic interactions. The antagonistic relationship between cadmium and zinc was observed in patients with degenerative changes. The correlation coefficient was −0.12 (Spearman’s correlation, *p* < 0.05). The correlation coefficients were significant in the cortical bone (−0.48), the cancellous bone (−0.47), and in the cancellous bone taken from the intertrochanteric area (−0.24) (Fig. 3).

The analysis of the relationship in the respective parts of the hip joint according to gender showed higher correlation coefficient values in men as compared with women (−0.59 and −0.46 in the cortical bone and −0.63 and −0.45 in the cancellous bone, respectively). Interactions between cadmium and zinc among smokers and non-smokers were noted only in the latter group (−0.14). Interestingly, no significant correlations with zinc were observed in the group of smokers despite additional cadmium intoxication.

## Discussion

In the current study, apart from the resected femoral head tissues, two other components were used for analysis: a fragment of the cancellous bone from the intertrochanteric area of the femoral bone and a component of the articular capsule. The part of the cancellous bone from the intertrochanteric area of the femoral bone is not directly affected by degenerative changes. However, the part of the articular capsule is made up of a totally different connective tissue which, theoretically, has no such accumulative capacity and at the same time may be influenced by degenerative processes.

Many authors, Jurkiewicz et al. [13], Wiechuła et al. [14], Kuo et al. [15], Brodziak-Dopierała et al. [16], Milachowski and Schramel [17], Garcia et al. [18], Lanocha et al. [19], Lanocha et al.[20], and Hisanaga et al. [21], have researched trace metals in the hip-building tissues. Jurkiewicz et al. [13] marked the content of cadmium in the cancellous bone of the femoral head in inhabitants of various regions of Poland. In the Silesia population, the content of cadmium was found to be 0.03 μg/g and that of zinc 84 μg/g. In our study, the levels of these elements were much higher (cadmium, 0.94 μg/g; zinc, 158.27 μg/g).

The content of cadmium in the cancellous bone of the femoral head in the population inhabiting northern Poland amounted to 0.035 mg/kg and was significantly lower when compared with our research [19]. The level of zinc in the population of northern Poland was 83.9 μg/g in the cancellous bone and 89.7 μg/g in the articular cartilage and the cortical bone. In our study, these values were 158.3 and 65.3 μg/g, respectively. This indicates significantly higher levels of the elements in the cancellous bone and lower in the articular cartilage and the cortical bone [20].

The levels of cadmium in bone tissues reported in literature were significantly different in the Spanish population 0.04 μg/g [18], in the contemporary Japanese population 0.5 μg/g [21], or contemporary inhabitants of Gran Canaria 0.52 μg/g [22]. Substantial differences in the content of this element were found in excavated samples dated back to various eras bc: 3.34–79.64 μg/g [21], 0.008–0.36 μg/g [23], 0.65–2.53 μg/g [22].

Kuo et al. [15], who studied bone tissue obtained from people living in highly industrialized Taiwan, estimated the content of cadmium at 1.2 μg/g and the content of zinc at 115 μg/g in the femoral head.

Researchers [1, 7, 8, 10, 14, 16, 23] have observed a greater tendency of cadmium to accumulate in women than in men. The comparative analysis of the levels of cadmium and zinc in the respective parts of the hip joint of men and women showed no significant differences. The levels of cadmium and zinc were higher in all bone components in men, except for the articular capsule where higher values were observed in women.

The antagonism of zinc and cadmium has been repeatedly described in literature [10, 15, 16]. It is believed that an increase in the content of cadmium results in a compensatory rise in zinc in the intoxicated tissues due to the binding of these metals with metallothionein. However, the interactions in small experimental animals differ from those observed in large mammals, including humans. They also vary depending on the type of tissue tested [12]. Intoxication of rats with cadmium ions causes a compensatory decrease in the content of zinc in the femoral bone [4, 11].

Smoking has a proven negative effect on the quality of bone tissue. Cadmium contained in tobacco smoke is one of the factors affecting the formation of osteoporosis [2, 24]. Tobacco was found to change the content of various elements, including zinc and cadmium, in tissues of the femoral head. Statistically significant differences were observed in the level of cadmium, but not in the level of zinc, between smokers and non-smokers (Mann Whitney *U* test, *p* < 0.01).

Jurkiewicz et al. [24], when examining the content of cadmium and zinc in the cancellous bone of the femoral head, found an increased content of zinc in non-smokers and a lower, but statistically insignificant, level of cadmium as compared with the smoking population (0.057 and 0.061 μg/g for cadmium and 84 and 81 μg/g for zinc, respectively).

Osteoarthritis is the most common indication for endoprosthetic surgery. Its etiology is usually difficult to determine, although it may partly result from anatomical and functional musculoskeletal disorders. Another reason for endoprosthetics is the femoral neck fracture. In the elderly, the fracture is mainly due to osteoporosis that affects the vertebral bodies and bases of the long bones, including femur. The analysis of the content of cadmium in groups with degenerative lesions and fractures showed a higher and statistically significant (Mann–Whitney *U* test, *p* < 0.025) level of this element in the group with fractures. These results confirm the long known effect of cadmium reducing bone mineral density and leading to osteoporotic fractures.

Milachowski and Schramel [17] investigated the femoral heads obtained from patients with degenerative changes and osteonecrosis. The statistical analysis showed a higher content of cadmium in samples with idiopathic necrosis of the femoral head when compared with the heads from degenerative joints and the control group [17].

Helliwell et al. [25] compared the content of zinc within the femoral heads from patients with fractures of the femoral neck and joints affected by degenerative processes. The content of zinc in the femoral heads was lower both in the samples obtained from patients with femoral neck fractures (154 μg/g) and those with degenerative processes (167 μg/g), as compared with the control group (205 μg/g) [25].

Antagonistic relationship between cadmium and zinc was observed in patients with degenerative changes, the correlation coefficient being −0.12 (Spearman’s correlation, *p* < 0.05). Significant correlation coefficients were found in the cortical bone (−0.48), the cancellous bone (−0.47), and the cancellous bone from the intertrochanteric area (−0.24). In the part of the hip joint which is not bone tissue, no significant antagonistic interactions were noted between cadmium and zinc. Correlation coefficients were higher in men as compared with women (0.59 in the cortical bone in men and −0.46 in women and −0.63 and −0.45 in the cancellous bone, respectively).

This type of correlation was found in women (−0.16) and in non-smokers (−0.14). Interestingly, in the group of smokers, no significant interactions with zinc were noted, despite additional cadmium intoxication.

Concluding, the highest levels of cadmium and zinc in the selected parts of the hip joint were found in the cancellous bone of the femoral head whereas the lowest in fragments of the articular capsule. This indicates a large cumulative capacity of bone tissue.

The patients with fractures showed a higher content of cadmium in comparison with patients with degenerative changes.

In the group of smokers, a higher content of cadmium was observed in comparison with non-smokers.

The content of zinc did not differ significantly between the population of smokers and non-smokers as well as patients with fractures and degenerative changes.

Antagonistic interrelationships between cadmium and zinc were found in tissues of the femoral head: the articular cartilage, the cortical bone, and the cancellous bone.
